# Early identification of atopic dermatitis patients in need of systemic immunosuppressive treatment

**DOI:** 10.1111/cea.13495

**Published:** 2019-09-30

**Authors:** Daphne S. Bakker, Julia Drylewicz, Stefan Nierkens, Edward F. Knol, Barbara Giovannone, Eveline M. Delemarre, Jorien van der Schaft, Deepak M. W. Balak, Marjolein S. de Bruin‐Weller, Judith L. Thijs

**Affiliations:** ^1^ Department of Dermatology and Allergology National Expertise Center for Atopic Dermatitis University Medical Center Utrecht Utrecht The Netherlands; ^2^ Laboratory of Translational Immunology University Medical Center Utrecht Utrecht The Netherlands

To the editor,

Atopic dermatitis (AD) is one of the most common chronic inflammatory skin diseases that are known to have profoundly negative effects on patient's quality of life.^1^ The majority of AD patients can be controlled with topical corticosteroids, but those with insufficient responses or who cannot reduce the potency/frequency of topical steroids to acceptable levels, will require treatment with systemic immunosuppressive/immunomodulating drugs. This group of patients can be defined as “difficult‐to‐treat” AD. In daily practice, the decision whether or not to start systemic therapy should be based on several factors, like disease severity, quality of life and comorbidities.^2^ A single severity measurement can, however, easily over‐ or underestimate the long‐term disease severity of a patient, since AD is characterized by exacerbations and remissions. Difficult‐to‐treat AD patients often experience a significant delay before optimal treatment is started. Early identification of this group might prevent unnecessary treatment delay. Therefore, the aim of this study was to construct a predictive serum biomarker signature, measured on a single time‐point, contributing to the separation between difficult‐to‐treat AD patients requiring systemic treatment and those who can be controlled with only topical therapy.

We retrospectively included 152 severe AD patients (median EASI score 28.8, IQR 25.3‐35.4; median age 32.0 years, IQR 22.0‐50.8; all Caucasian) from the National Expertise Center for AD in the Netherlands, who were initially inadequately treated with topical corticosteroids. Subsequently, all patients started with intensive topical treatment and defined as the use of at least six weeks of daily treatment with high amounts of potent topical corticosteroids after adequate training and instructions in self‐management. Patients with physician reported doubts on treatment compliance were excluded. During this treatment period, 74 severe AD patients (EASI > 21 before start of treatment) could be controlled with topical steroids (“controlled disease” group), and 78 severe AD patients (EASI > 21 before start of treatment) eventually required treatment with systemic immunosuppressive drugs (“difficult‐to‐treat” group; Table 1). Serum was collected before start of intensive topical treatment, and 129 serum biomarkers (Table S1), measured using Luminex‐based multiplex immunoassays, were included for analysis. To construct the prognostic biomarker signature, we used a statistical algorithm previously developed by Mamtani et al^3^ (detailed methods related to patient and sample selection, serum biomarker measurements and statistical analysis are available in the article's Online Appendix S1).

**Table 1:** Patient characteristics

Clinical characteristics	Group 1: Controlled disease (n = 74)	Group 2: Difficult to treat (n = 78)	*P*‐value differences
Age (years)[Link], median [IQR]	29.0 [22.0 ‐ 48.3]	37.0 [22.0 ‐ 52.3]	.522[Link]
Male, n (%)	38 (51%)	47 (60%)	.269[Link]
EASI score, median [IQR]	27.8 [24.7 ‐ 31.5]	29.8 [25.3 ‐ 39.0]	**.038** [Link]
Atopic diseases, n (%)			
Allergic asthma	40 (54%)	43 (51%)	.756[Link]
Allergic rhinitis	47 (64%)	51 (65%)	.611[Link]
Food allergy	26 (35%)	35 (45%)	.230[Link]
No other atopic disease besides AD	15 (20%)	13 (17%)	.592[Link]
Missing data	0	3 (4%)	
Age of onset, n (%)			
0‐1 years	30 (41%)	29 (37%)	.370[Link]
2‐11 years	30 (41%)	38 (49%)	
12‐18 years	3 (4%)	1 (1%)	
>18 years	4 (5%)	7 (9%)	
Missing data	7 (10%)	3 (4%)	
Hospitalization for AD (after study inclusion and sampling), n (%)	27 (36.5%)	44 (56%)	**.036** [Link]

Note

Categorical variables are presented as counts and percentages; continuous variables are presented as median [IQR].

Abbreviations: EASI Eczema Area Severity Intensity; IQR interquartile range; VAS visual analogue scale.

Age at time of sample collection.

Wilcoxon rank‐sum test

Chi‐square test, p‐value < 0.05 was considered statistically significant.

Bold values are used to highlight significant differences.

Stepwise multiple regression analysis resulted in the selection of eight serum biomarkers, including interleukin (IL)‐1b, platelet factor 4 (PF4/CXCL4), cutaneous T cell‐attracting chemokine (CTACK/CCL27), Trappin‐2, Sclerostin (SOST), gamma‐tubulin complex protein 2 (GCP‐2), soluble programmed death‐1 (sPD‐1) and leucocyte associated immunoglobulin‐like receptor‐1 (LAIR‐1), which were combined by using a linear discriminant function analysis to construct the final prediction model for classification of patients into “controlled disease” or “difficult to treat”. The final model had an *R* ^2^ of 0.70, a Wilk's λ of 0.51 and predicted the classification correctly in 125 (82%) out of the 152 patients. Sixteen patients were misclassified as controlled disease, and eleven patients were misclassified as difficult to treat, resulting in a sensitivity of 78%, a specificity of 86%, a positive predictive value (PPV) of 84% and a negative predictive value (NPV) of 81%. CTACK was the best individual predicting biomarker (AUC value of 0.73). After combining the eight biomarkers, the AUC of the final prediction model raised to 0.89 (Figure 1A and 1).

**Figure 1:**
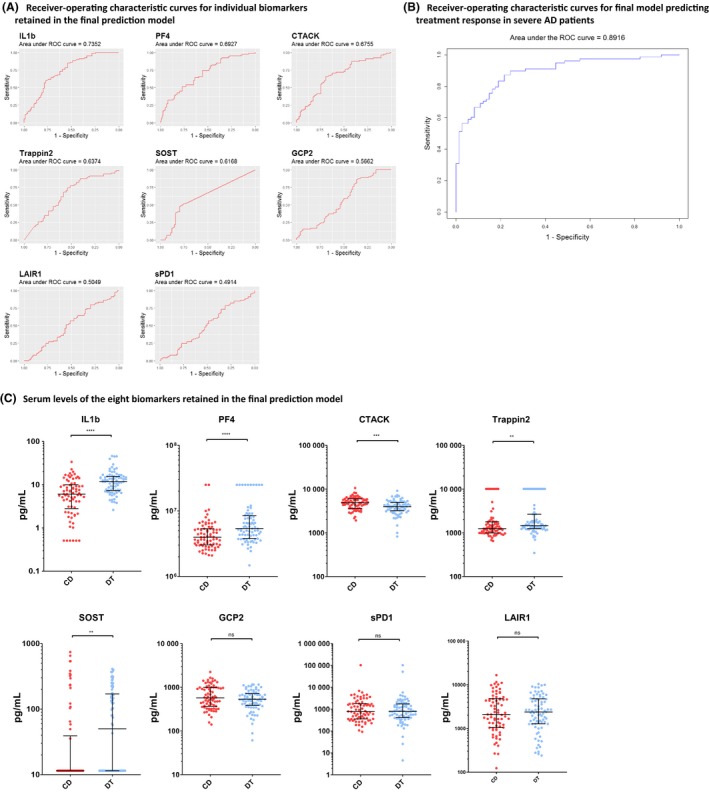
Receiver‐operating characteristics and serum levels for individual biomarkers and the final model predicting treatment response in severe AD patients. A, Individual ROC curves for interleukin 1 beta (IL‐1b), platelet factor 4 (PF4), cutaneous T cell‐attracting chemokine (CTACK), transglutaminase substrate and WAP domain‐containing protein/Elafin (Trappin‐2), Sclerostin (SOST), gamma‐tubulin complex protein 2 (GCP‐2), soluble programmed death protein 1 (sPD‐1) and leucocyte associated immunoglobulin‐like receptor 1 (LAIR‐1), which were retained in step 2 of the statistical algorithm. B, Receiver‐operating characteristic (ROC) curve for final model which included IL‐1b, PF4, CTACK, Trappin‐2, SOST, GCP‐2, sPD‐1 and LAIR‐1. Combining these eight biomarkers in a biomarker signature increased the capacity to predict treatment responses in severe AD patients. C, Differences in serum biomarker levels between severe AD patients who can be controlled with topical corticosteroids (“controlled disease”, CD) and patient who require treatment with systemic immunosuppressive drugs (“difficult to treat”, DT) were compared using Mann‐Whitney U tests. Horizontal bars represent median biomarker levels with interquartile range. *P < .05; ** *P* < .01; *** *P* < .001; ns, non‐significant

Serum levels of IL‐1b, PF4/CXCL4, Trappin‐2 and SOST were significantly higher in the difficult‐to‐treat group compared to the controlled disease patients (Figure 1C). Serum levels of CTACK were significantly higher in the controlled disease patients. No differences were found in levels of GCP‐2, sPD‐1 and LAIR‐1. However, these three biomarkers significantly improved the prediction capacity of the final model.

Of the eight identified biomarkers, four have previously been shown to contribute to chronic skin inflammation or AD pathogenesis. PF4/CXCL4 and CTACK/CCL27 are higher expressed in serum of AD patients compared to healthy controls and correlate with AD severity.^4^, ^5^Levels of PF4 were, correspondingly, significantly higher, whereas levels of CTACK were significantly lower in our difficult‐to‐treat group, in which median EASI score was significantly higher (29.8, IQR 25.3‐39.0 versus 27.8, IQR 24.7‐31.5 in the controlled disease group). However, this small absolute difference in disease severity is not considered to be clinically relevant.^6^ Despite PF4 and CTACK have been found to correlate with AD disease severity, both markers are considered not to be the optimal markers to pick up a small difference in disease severity. Serum levels of thymus and activation‐regulated chemokine (TARC/CCL17), currently the best performing biomarker for assessing disease severity in AD,^5^ did not significantly differ between the two groups and was not included in the final model, indicating that the current model is not solely based on differences in disease severity based on a single EASI score, but may reflect the more long‐term disease severity and treatment response. IL‐1b is a pro‐inflammatory cytokine, which can induce IL‐20 production and thereby keratinocyte differentiation.^7^, ^8^ Gamma‐tubulin complex protein 2 (GCP‐2) is a chemoattractant for neutrophilic granulocytes and has shown to be up‐regulated by IL‐4, one of the main contributors to the pathogenesis of AD.^9^

The role of the four remaining biomarkers in the pathogenesis of AD has not been explored yet. This is the first study investigating these markers in a large cohort of AD patients. Trappin‐2, SOST, sPD‐1 and LAIR‐1 have all been associated with immune regulation, and might thus play a role in AD pathogenesis. Our results imply that pathophysiological heterogeneity in immunological pathways might underlie differences in treatment responses, and may be used to distinguish a specific subpopulation of difficult‐to‐treat AD patients in need of systemic treatment from patients who can be controlled with topical therapy.

In the current study, patients were stratified based on treatment history necessary to control the AD. The decision whether or not to start systemic therapy in AD patients is not always easy; several factors need to be considered.^2^ The lack of response to adequately applied topical treatment or long‐term need of large amounts of topical steroids is a very important indicator for systemic treatment, taken into consideration that much effort should be made to optimize topical treatment. In all included patients, much attention was paid to adherence to topical treatment and evaluation of self‐management. However, treatment compliance to topical therapy cannot be fully guaranteed.

With the correlation coefficient, NPV and PPV of the final model appearing to be sub‐optimal, a potential danger of using this predictive signature in clinical practice might be unnecessary treatment with systemic immunosuppressive drugs due to incorrectly assigning a patient as “difficult‐to‐treat”. Hence, we do not aim to replace clinical decision making by our biomarker signature. Instead, this signature might serve as a valuable addition to the decision whether or not to start systemic therapy in individual AD patients and might accelerate the initiation of optimal therapy. Validation of our biomarker signature in a prospective patient population is necessary to evaluate its applicability and predictive capacity.

In conclusion, this study shows that a constructed predictive signature of eight serum biomarkers is able to identify a subgroup of severe, difficult‐to‐treat AD patients with a sensitivity of 78% and a specificity of 86%, which might contribute to earlier identification. This signature might serve as a valuable addition to the decision whether to start systemic therapy or not in individual AD patients, and the statistical algorithm used in this study may also be applied to construct biomarker signatures predicting treatment response to systemic immunosuppressive drugs, dupilumab or other therapies in the future. Since more targeted therapies will play an increasingly important role in AD treatment, prediction of treatment response can significantly contribute to selecting the right treatment for the right patient.

## CONFLICT OF INTEREST

MS de Bruin‐Weller: consultant for Regeneron Pharmaceuticals, Inc, Sanofi Genzyme; an advisory board member for AbbVie, Regeneron Pharmaceuticals, Inc, Sanofi Genzyme; and principal investigator for AbbVie, Regeneron Pharmaceuticals, Inc, Roche, Sanofi Genzyme. The other authors have no conflicts of interest to declare.

## Data Availability

The data that support the findings of this study are available from the corresponding author upon reasonable request.

## References

[1] Eckert L , Gupta S , Amand C , et al. Impact of atopic dermatitis on health‐related quality of life and productivity in adults in the United States: an analysis using the national health and wellness survey. J Am Acad Dermatol. 2017;77(2): 274‐279.e3. 28606711 10.1016/j.jaad.2017.04.019

[2] Simpson EL , Bruin‐Weller M , Flohr C , et al. When does atopic dermatitis warrant systemic therapy? Recommendations from an expert panel of the International Eczema Council. J Am Acad Dermatol. 2017;77(4):623‐633. 10.1016/j.jaad.2017.06.042 28803668

[3] Mamtani MR , Thakre TP , Kalkonde MY , et al. A simple method to combine multiple molecular biomarkers for dichotomous diagnostic classification. BMC Bioinformatics. 2006;7:442. 17032455 10.1186/1471-2105-7-442 PMC1618410

[4] Tamagawa‐Mineoka R , Katoh N , Ueda E , et al. Elevated platelet activation in patients with atopic dermatitis and psoriasis: increased plasma levels of beta‐thromboglobulin and platelet factor 4. Allergol Int. 2008;57(4):391‐396. 18797178 10.2332/allergolint.O-08-537

[5] Thijs J , Krastev T , Weidinger S , et al. Biomarkers for atopic dermatitis: a systematic review and meta‐analysis. Curr Opin Allergy Clin Immunol. 2015;15(5):453‐460. 26226355 10.1097/ACI.0000000000000198

[6] Schram ME , Spuls PI , Leeflang MM , et al. EASI, (objective) SCORAD and POEM for atopic eczema: responsiveness and minimal clinically important difference. Allergy. 2012;67(1):99‐106. 21951293 10.1111/j.1398-9995.2011.02719.x

[7] Otkjaer K , Kragballe K , Johansen C , et al. IL‐20 gene expression is induced by IL‐1beta through mitogen‐activated protein kinase and NF‐kappaB‐dependent mechanisms. J Invest Dermatol. 2007;127(6):1326‐1336. 10.1038/sj.jid.5700713 17255956

[8] Clarysse K , Pfaff CM , Marquardt Y , et al. JAK1/3 inhibition preserves epidermal morphology in full‐thickness 3D skin models of atopic dermatitis and psoriasis. J Eur Acad Dermatol Venereol. . 2019;33(2):367‐375. 30357932 10.1111/jdv.15301

[9] Bao L , Shi VY , Chan LS . IL‐4 up‐regulates epidermal chemotactic, angiogenic, and pro‐inflammatory genes and down‐regulates antimicrobial genes in vivo and in vitro: relevant in the pathogenesis of atopic dermatitis. Cytokine. 2013;61(2):419‐425. 23207180 10.1016/j.cyto.2012.10.031

