# Supplementary material for: Early identification of atopic dermatitis patients in need of systemic immunosuppressive treatment
Source: Clin Exp Allergy. 2019 Sep 30;49(12):1641–4. doi: 10.1111/cea.13495 (PMC6973172; doi:10.1111/cea.13495)
Supplement: Supplementary file 1 [file CEA-49-1641-s001.docx]

**ONLINE SUPPLEMENTARY MATERIAL**

**Early identification of atopic dermatitis patients in need of systemic immunosuppressive treatment**

Daphne S. Bakker^1^, MD; Julia Drylewicz^2^, PhD; Stefan Nierkens^2^, PhD; Edward F. Knol^1,2^, PhD; Barbara Giovannone^2^, PhD; Eveline M. Delemarre^2^, MD, PhD; Jorien van der Schaft^1^, MD, PhD; Deepak M.W. Balak^1^, MD, PhD; Marjolein S. de Bruin-Weller^1^, MD, PhD; Judith L. Thijs^1^, MD, PhD

^1^ National Expertise Center for Atopic Dermatitis, Department of Dermatology and Allergology, University Medical Center Utrecht, The Netherlands
^2^ Laboratory of Translational Immunology, University Medical Center Utrecht, Utrecht, The Netherlands

**Corresponding author:**

Daphne Bakker, M.D.

University Medical Centre Utrecht, Department of Dermatology

Heidelberglaan 100, 3584 CX

Utrecht, The Netherlands

E-mail: [d.s.bakker-4@umcutrecht.nl](mailto:d.s.bakker-4@umcutrecht.nl)

Telephone number: +31 887573933

**METHODS**

*Patients and samples*

Two groups of severe AD patients (EASI > 21), as defined by the criteria of Hanifin and Rajka^1^, were included retrospectively from our AD database, including all AD patients treated at our center, who have given written informed consent for the use of the data recorded in their electronic medical records. Electronic medical records were manually screened and patients were stratified based on their treatment history necessary to control the eczema. Multiple severity measurements over time were used to define these groups. Following our local treatment protocols, all patients were initially treated with intensive topical treatment, defined as the use of at least six weeks of daily treatment with high amounts of potent topical corticosteroids after adequate training and instructions. Patients with physician reported doubts on treatment compliance were excluded. Group 1 consisted of patients with severe AD (EASI >21), who could be “controlled” with topical corticosteroids (controlled disease). Controlled disease was defined as an EASI score of 7 or less. Group 2 consisted of patients with severe AD (EASI >21) who needed systemic treatment to control their AD (difficult to treat). Group 2 included (a) patients with uncontrolled severe AD despite intensive topical treatment, or (b) patients with severe disease experiencing exacerbations upon tapering of topical treatment to safe maintenance schedules, who are therefore in need of systemic (immunosuppressive) therapy. Patients who were treated with oral immunosuppressive drugs or UV-light therapy within three months before sampling were excluded. The following data were retrospectively retrieved from the patient’s electronic medical files: sex, age, EASI score at moment of sampling, history of asthma, allergic rhinitis, allergic conjunctivitis and food allergy, age of onset of AD and history of hospitalization for AD. Serum samples were routinely collected before start of treatment during uncontrolled disease (EASI>21) and stored at -80 degrees Celsius in a biobank until analysis.

The protocol used in this study was approved by the Institutional Review Board of the University Medical Center Utrecht, adhering to the Declaration of Helsinki Principles.

*Power calculation*

The sample size (n) needed for this study was determined using a frequentist approach. The disease status aimed to predict in this study applies to a binominal test; having “difficult to treat” AD or not. This will give a sequence of 0's and 1's which are called ‘x’. The entries of ‘x’ will have a Bernoulli distribution with success probability ‘p’. The estimate of ‘p’ is given by p =∑x/n.

In order to answer the question how big ‘n’ should be, additional information on biomarkers as disease predictors in AD is needed. The best known biomarker for disease severity in AD is serum thymus and activation-regulated chemokine (TARC). Serum TARC levels have been determined in relatively large numbers of patients from different studies, showing pooled correlation coefficients of 0.60 (95% CI 0.48–0.70) and 0.64 (95% CI 0.57–0.70) in longitudinal and cross-sectional studies, respectively.^2^ To calculate the sample size we used a sensitivity cut-off value of 0.60 and a power of 0.80, so there is an 80% chance to detect a sensitivity of p=0.60, with a significance level controlled of α=0.05. By using the “pwr” package in R.^3^ we calculated a sample size of 152 AD patients (effect size=0.21) needed to match our required sensitivity.

*Serum protein biomarker analysis*

A panel of 143 serum biomarkers (all markers currently available in our center) were measured using Luminex technology at the Multiplex Core Facility of the Laboratory for Translational Immunology (UMC Utrecht, The Netherlands), using an in-house validated panel of analytes, listed in Table S1. Uniquely color-coded magnetic beads (MagPlex Microsperes, Luminex, Austin, Texas) were conjugated to antibodies specific for the reported analytes and incubated with 50 µL of standard dilutions per sample for 1 hour (continuous shaking in the dark). Samples were diluted in High Performance Elisa buffer (HPE; Sanquin, The Netherlands). Pre-treatment of samples included filtration and incubation with HeteroBlock to prevent interference by binding of heterophilic antibodies. Plates were washed (Bio-Plex Pro II Wash Station; Bio-Rad, Hercules, California, USA) and a corresponding cocktail of biotinylated detection antibodies was added for 1 hour. Repeated washings were followed by a 10 minute streptavidin-phycoerythrin (PE) incubation. Fluorescence intensity of PE was measured using a Flexmap 3D system (Luminex) and analyzed by using BioPlex Manager software version 6.1; (Bio-Rad) using 5-parameter curve fitting.^4^ Serum biomarkers with signals above or below the assay detection limit in >60% of the samples were excluded for further analyses, resulting in 129 unique serum biomarkers selected for further analysis.

*Statistical analysis*

Data were analyzed using R version 3.4.1 (R Foundation for Statistical Computing, Vienna, Austria) and the SPSS (Statistical Package for the Social Science) software for Windows version 21.0 (SPSS Inc., Chicago, IL, USA). Differences in clinical characteristics between the two patient groups were compared using the Wilcoxon rank sum test for continuous variables, and with the chi-square test for categorical variables. P-values lower than 0.05 were considered statistically significant.

Serum samples that were above or below the assay limits of detection were given values equivalent to the lower limit divided by two or the upper limit multiplied by two. Actual concentration data were normalized by a log-transformation.

*Prognostic biomarker signature*

To construct the prognostic biomarker signature, we used the method that has been previously developed by Mamtani et al^5^. This method consists of three steps: 1. screening the biomarkers individually based on the Performance Index (Pi) which is a function of the estimated area under the receiver characteristic curve (AUC); 2. using stepwise multiple regression analysis to select the top ranked n-1 biomarkers; 3. combining the selected biomarkers using a linear discriminant function (Figure 1). In the stepwise multiple regression analysis, a retention criterion of 0.01 was used to define if a biomarker should be kept in the multivariate model. The best model was assessed by its R^2^ and its complement Wilks’ λ. The Wilks’ λ is a measure of how well the model separates the cases into groups. Smaller values of Wilks’ λ indicate greater discriminatory ability of the model.

Posterior probabilities from the linear discriminant function analysis were used to define a predicted classification (group 1: “controlled disease” or group 2: “difficult to treat”) for each individual. If the posterior probability for a given patient was higher for group 1 than for group 2 the predicted classification was defined as group 1, and vice versa. Sensitivity, specificity, positive predictive value (PPV) and negative predictive value (NPV) of the final prediction model were calculated based on predicted and observed classifications.

**REFERENCES**

1. Hanifin JM, Rajka G. Diagnostic Features of Atopic-Dermatitis. *Acta Derm-Venereol* 1980:44-47.

2. Thijs J, Krastev T, Weidinger S, et al. Biomarkers for atopic dermatitis: a systematic review and meta-analysis. *Curr Opin Allergy Clin Immunol* 2015;15(5):453-60. doi: 10.1097/ACI.0000000000000198

3. pwr CS. Basic Functions for Power Analysis. R package version 1.2-2.

4. Thijs JL, Strickland I, Bruijnzeel-Koomen C, et al. Moving toward endotypes in atopic dermatitis: Identification of patient clusters based on serum biomarker analysis. *J Allergy Clin Immunol* 2017;140(3):730-37. doi: 10.1016/j.jaci.2017.03.023 [published Online First: 2017/04/17]

5. Mamtani MR, Thakre TP, Kalkonde MY, et al. A simple method to combine multiple molecular biomarkers for dichotomous diagnostic classification. *BMC Bioinformatics* 2006;7:442. doi: 10.1186/1471-2105-7-442 [published Online First: 2006/10/13]

**SUPPLEMENTARY TABLES**

**Table S1. List of serum biomarkers measured by using Luminex technology**

| Serum biomarkers measured via Luminex technology | | | | |
| --- | --- | --- | --- | --- |
| IL-1a | I-309 | G-CSF | Trappin-2 | Cat L |
| IL-1b | MCP-1 | SCF | Endoglin | Cat S |
| IL-3 | MIP-1a | HGF | TIM/KIM-1 | TACI |
| IL-4 | MIP-1b | EGF | SDF-1a | Gal-3 |
| IL-5 | MCP-3 | FGF Basic | DKK1 | C5a |
| IL-6 | MCP-2 | NGF | Apelin | ACE |
| IL-7 | Eotaxin | PIGF | S100A8 | PF-4 |
| IL-9 | MCP-4 | VEGF | Gal-9 | NAP-2 |
| IL-10 | TARC | TREM-1 | Ang-1 | Adiponectin |
| IL-12 | MIP-3b | Cat B | Ang-2 | MMP-9 |
| IL-13 | MIP-3a | sPD-1 | Tie-2 | sCD14 |
| IL-15 | MDC | FAS | YKL-40 |  |
| IL-18 | MPIF | FAS-L | LAP | **Excluded for analysis** |
| IL-20 | TECK | LAIR-1 | RANTES | *IL-1RA* |
| IL-21 | Eotaxin-3 | IL-18BPa | PARC | *IL-2* |
| IL-22 | C-TACK | IL-1R1 | Adipsin | *IL-11* |
| IL-23 | Gal-9 | IL-1R2 | Leptin | *IL-17* |
| IL-26 | GRO-1a | ST-2 |  | *IL-25* |
| IL-27 | ENA-78 | TNF-R1 | Resistin | *IL-29* |
| IL-31 | GCP-2 | TNF-R2 | PAI-1 | *IL-37* |
| IL-33 | IL-8 | sIL-2R | RBP4 | *IFNg* |
| TNFa | MIG | sCD163 | TPO | *LIF* |
| TNFb | IP-10 | sVEGF-R1 | SAA-1 | *M-CSF* |
| IFNa | I-TAC | sSCF-R | BDNF | *GM-CSF* |
| IFNb | BLC | Gal-1 | sICAM | *EPO-R* |
| LIGHT | BRAK | P-sel | sVCAM | *Chemerin* |
| TWEAK | XCL-1 | E-sel | MMP-1 | *Chemerin* |
| MIF | OPG | Cystatin C | MMP-3 |  |
| OSM | OPN | SLP1 | MMP-8 |  |
| TSLP | SOST | Elastase | TIMP-1 |  |

This table shows all 143 serum biomarkers that were measured by using Luminex-based multiplex immunoassays in 152 severe AD patients. 14 biomarkers were excluded for further analyses due to more than 60% of the samples being above or below the assay detection limits.
